# The Molecular Switch of Telomere Phages: High Binding Specificity of the PY54 Cro Lytic Repressor to a Single Operator Site

**DOI:** 10.3390/v7062746

**Published:** 2015-06-02

**Authors:** Jens Andre Hammerl, Nicole Roschanski, Rudi Lurz, Reimar Johne, Erich Lanka, Stefan Hertwig

**Affiliations:** 1 Bundesinstitut für Risikobewertung (Federal Institute for Risk Assessment), Department of Biological Safety, Diedersdorfer Weg 1, D-12277 Berlin, Germany; E-Mails: jens-andre.hammerl@bfr.bund.de (J.A.H.); reimar.johne@bfr.bund.de (R.J.); 2 Free University Berlin, Institute of Animal Hygiene and Environmental Health, Robert-von-Ostertag-Str. 7-13, D-14163 Berlin, Germany; E-Mail: nicole.roschanski@fu-berlin.de; 3 Max-Planck-Institut für Molekulare Genetik, Ihnestraße 63-73, D-14195 Berlin, Germany; E-Mails: lanka@molgen.mpg.de (E.L.); lurz@molgen.mpg.de (R.L.)

**Keywords:** PY54, N15, telomere phages, molecular switch, Cro, lytic repressor, DNA binding

## Abstract

Temperate bacteriophages possess a molecular switch, which regulates the lytic and lysogenic growth. The genomes of the temperate telomere phages N15, PY54 and ϕKO2 harbor a primary immunity region (*immB*) comprising genes for the prophage repressor, the lytic repressor and a putative antiterminator. The roles of these products are thought to be similar to those of the lambda proteins CI, Cro and Q, respectively. Moreover, the gene order and the location of several operator sites in the prototype telomere phage N15 and in ϕKO2 are also reminiscent of lambda-like phages. By contrast, *in silico* analyses revealed the presence of only one operator (O_R_3) in PY54. The purified PY54 Cro protein was used for EMSA studies demonstrating that it exclusively binds to a 16-bp palindromic site (O_R_3) upstream of the prophage repressor gene. The O_R_3 operator sequences of PY54 and ϕKO2/N15 only differ by their peripheral base pairs, which are responsible for Cro specificity. PY54 *cI* and *cro* transcription is regulated by highly active promoters initiating the synthesis of a homogenious species of leaderless mRNA. The location of the PY54 Cro binding site and of the identified promoters suggests that the lytic repressor suppresses *cI* transcription but not its own synthesis. The results indicate an unexpected diversity of the growth regulation mechanisms in lambda-related phages.

## 1. Introduction

**Figure 1:**
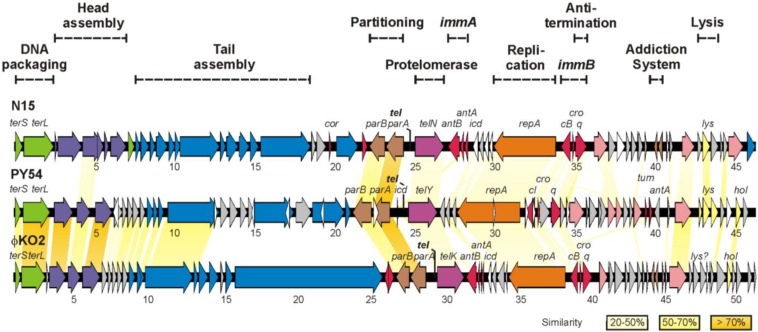
Genome organization of the phages N15, PY54 and ϕKO2. Colors indicate the predicted functions of gene products. Related genes of the phages are connected by colored shading.

Within the lambdoid phage family, the temperate phages N15, PY54 and ϕKO2 isolated from *E. coli*, *Yersinia (Y.) enterocolitica* and *Klebsiella (K.) oxytoca*, respectively, belong to a particular subgroup, as their prophages are linear plasmids with covalently closed hairpin ends. The genomes of these so-called telomere phages have been thoroughly characterized [1,2,3]. While N15 and PY54 have a nearly identical genome size of 46.3 kb, the ϕKO2 genome is larger (51.6 kb). Sequence analyses disclosed that the left arm of PY54, which mainly contains virion structural genes is more closely related to ϕKO2 than to N15, whereas N15 and ϕKO2 show the strongest homologies in the right arm harboring genes involved in e.g., the generation and replication of the linear plasmid, phage immunity and host cell lysis (Figure 1). *In vivo* observations showed that the N15 and ϕKO2 plasmid prophages belong to the same incompatibility group, whereas the PY54 prophage belongs to a separate group and thus is compatible with the two other plasmids [4].

**Figure 2:**
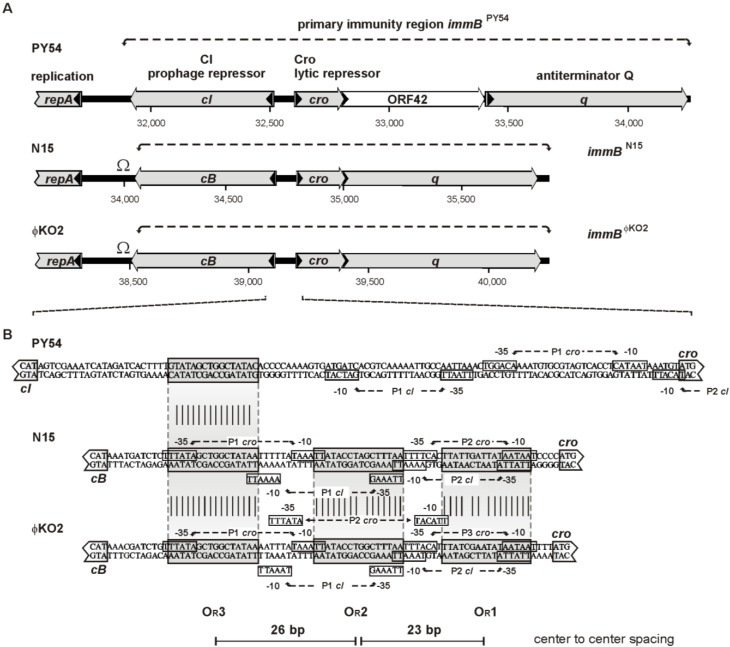
Organization of the primary immunity region (*immB*) of the linear plasmid prophages PY54, ϕKO2 and N15. (**A**) Genetic map of *immB*. Functional assignments were made according to Hertwig *et al.* [13], Casjens *et al.* [1], and Ravin *et al.* [14]. The transcription directions are indicated by arrows, Ω, transcriptional terminators identified in ϕKO2 and N15; (**B**) Sequence alignment of the intergenic regions between the repressor genes *cI* (*cB*) and *cro*. Operator sequences of N15 determined by Lobocka *et al.* [6] and similar sequences in the corresponding regions of PY54 and ϕKO2 are presented in light shaded boxes. Nucleotides within the PY54 and ϕKO2 operator sequences identical to those of the corresponding N15 sites are marked by vertical lines. Predicted promoters of *cI* and *cro* are shown.

Like other temperate phages, the telomere phages possess genes providing the molecular switch between the lytic and the lysogenic cycle and important functions for prophage induction. N15 induction is initiated by the expression of *antC* whose product acts against the N15 prophage repressor [5]. For the switch between the lytic and lysogenic cycle, two loci termed primary immunity region (*immB*) and secondary immunity region (*immA*) have been identified [6,7]. The *immA* locus, which is an operon in N15 and ϕKO2, comprises three genes coding for the repressor AntA, an inhibitor of cell division, Icd, and a protein denoted AntB whose function has not been elucidated as yet. In PY54 an *antB* gene is missing and *antA* and *icd* are widely separated on the right arm of the phage genome (Figure 1). The *immB* locus of the telomere phages is comparable to the immunity region of lambda-like phages but exhibits a simpler arrangement. It encodes products related to the prophage repressor CI (in N15 and ϕKO2 CB), lytic repressor Cro and a putative antiterminator Q as well as operator sites (O_R_) located between *cI* and *cro* (Figure 2). In lambda, both CI and Cro bind to the three related operator sites O_R_1_,_ O_R_2 and O_R_3, but with reversed affinity [8]. The prophage repressor has a strong binding affinity for O_R_1 and O_R_2_._ Since these operators overlap the *cro* promoter P_R_, binding of CI to O_R_1 and O_R_2 leads to a repression of *cro* transcription. Additionally, CI activates its own expression by activating the RNA polymerase. In this way, lysogeny is established and maintained. At high concentrations, CI may also bind to O_R_3 resulting in the repression of its own synthesis. Conversely, Cro has the highest affinity for O_R_3 overlapping the *cI* promoter P_RM_ As a consequence, *cI* transcription is blocked and the lytic cycle is induced. As with CI, Cro may also autoregulate its own expression by binding to O_R_1. Compared to lambda, little is known on the genetic switch of telomere phages. In N15 and PY54, the function of the prophage repressors has already been demonstrated and binding sites for the N15 CB repressor have been determined [2,6]. Similar to lambda, binding was observed to three operators (Figure 2B) situated between *cB* and *cro*. In addition, two operators (O_L_) overlapping with the predicted promoter of the plasmid replication gene *repA* were bound [9]. N15 CB was therefore suggested to be implicated in both the regulatory circuitry of phage propagation and the control of plasmid replication [10]. Contrary to the prophage repressors, there is a lack of information about the activity of the Cro proteins of the telomere phages. Dubrava *et al.* [11] studied the structure and dimerization of the N15 Cro protein in comparison to lambda Cro. Similarities to lambdoid phages enabled Hall *et al.* [12] to predict amino acid residues of the recognition helices of N15 Cro and base pairs within operator half-sites probably involved in binding to the repressor. Due to the close relationship between N15 and ϕKO2, Casjens *et al.* [1] suggested the same repressor target specificity for these phages. However, experimental data on this issue are missing. Lytic repressor activity of the telomere phages has been demonstrated only for Cro of PY54 [13] but it is not known to which sequences on the PY54 genome the protein binds and whether it may recognize operator sites of N15 and ϕKO2. Unlike N15 and ϕKO2, only one operator site (O_R_3) adjacent to *cI* has been identified in PY54 by *in silico* analysis (Figure 2), suggesting that the genetic switch of this phage may diverge.

In this work, we studied the activity and binding specificity of the PY54 Cro repressor under *in vivo* and *in vitro* conditions. We show that the protein exerts repressor activity and that it is highly specific with respect to its DNA target. EMSA studies with the purified Cro repressor revealed binding to a single site on the PY54 genome, adjacent to the prophage repressor gene but no binding to N15 and ϕKO2 DNA. Using luciferase assays, several very strong promoters were identified in the PY54 *cro* region, which are responsible for the initiation of the synthesis of leaderless messenger RNAs.

## 2. Materials and Methods

### 2.1. Bacterial Strains, Plasmids and Growth Conditions

All strains and plasmids used in this study are listed in Table S1. Phage PY54 was isolated from *Y. enterocolitica* strain 29854 [2,15,16,17]. The *E. coli* strain Genehogs (Invitrogen, Karlsruhe, Germany) was used for cloning procedures. *E. coli* strain SCS1 (Stratagene, Amsterdam, Netherlands) was used as host for the overexpression of the PY54 *cro* gene. PY54 propagation was performed in *Y. enterocolitica* 83/88/2 [4,13]. Besides this strain which was cultivated at 28 °C, strains of *E. coli* were incubated at 37 °C. If not stated otherwise, all strains were grown in Luria Bertani (LB)-broth [18]. Solid media contained 1.8% (*w*/*v*) agar. When required, ampicillin and kanamycin were supplemented at 100 µg·mL^−1^ and chloramphenicol and tetracycline at 12.5 µg·mL^−1^.

### 2.2. Bacteriophages and Construction of Phage Mutants

PY54, N15 and ϕKO2 have been characterized by Hertwig *et al.* [2,13,19], Ravin *et al.* [3,14], and Casjens *et al.* [1], respectively. The construction of the N15-D04 (Cm^r^) mutant has been previously described [4]. PY54-35Tc was generated by inserting the tetracycline resistance gene (*tetA*) of Tn5 into ORF35 of PY54 (position 27,463; upstream of *repA*). This was accomplished by introducing an EcoRI fragment of PY54 (positions 26,297 to 28,429) into pLitmus38 (Ap^r^; NEB). The recombinant plasmid contained a unique NruI restriction site within the coding sequence of ORF35. The *tetA* gene of Tn5 was amplified by PCR using plasmid pBR329 (Ap^r^, Tc^r^, Cm^r^) [20] as template, and then inserted into the NruI restriction site of ORF35. Following this, the resulting plasmid was introduced into *Y. enterocolitica* 83/88/2. After propagation of PY54 on the transformants, recombinant phages were selected by lysogenization of strain 83/88/2 on CIN agar [21] supplemented with tetracycline. Mutant PY54-35Tc was shown to have a phenotype similar to the wild type phage.

### 2.3. In Vivo Assay for the PY54 Cro Repressor Activity

To study the influence of the repressor on PY54 and N15 propagation, a plasmid containing the PY54 *cro* gene and its putative promoter sequences was constructed. The *cro* gene including contiguous sequences up to *cI* was amplified by PCR using purified phage DNA as template. The forward and reverse primers contained embedded restriction sites for XbaI and HindIII, respectively. After digestion with XbaI and HindIII (Biolabs, Frankfurt am Main, Germany), the fragment was inserted into the corresponding sites of pIV2 (Km^r^) [22]. Upon transformation of *E. coli* strain Genehogs, the recombinant plasmid pJH192 (Table S1) was isolated and verified by sequencing. For *in vivo* experiments, the plasmid was introduced into *E. coli* C-1a and *Y. enterocolitica* 83/88/2. Strains were grown to a density at A_588_ of 1.0. Thereafter, a 100 µL aliquot of a diluted phage lysate and 100 µL of the indicator strains were incubated for 10 min at room temperature. Phage titers (PFU) were determined by the standard soft agar overlay method [18]. The efficiency of plating (EOP) was determined by plating infected bacteria on agar containing tetracycline and kanamycin (PY54-35Tc) or chloramphenicol and kanamycin (N15-D04). The PY54 and N15 phage lysates were prepared by mitomycin C (2.5 µg/mL) induction under conditions as previously described [4].

### 2.4. Overproduction and Purification of the Repressor Protein

DNA of CsCl-purified phage particles of PY54 was used as template for the PCR amplification of the *cro* gene. Forward and reverse primers were deduced from the published PY54 sequence (GenBank accession no. AJ564013) [13] and contained embedded restriction sites for NdeI and HindIII, respectively. The PCR product was digested with these enzymes and inserted into the corresponding sites of pMS470Δ8*cat* [4,23] yielding pJH142-2 (Table S1).

Plasmid pMS470Δ8*cat* contains the *tac* promoter and *lacI_q_* gene to control gene expression. Following transformation of an *E. coli* strain with the respective plasmid, *cro* expression was induced with isopropyl-1-thio-β-d-galactopyranoside (IPTG). Bacteria were grown under shaking conditions (180–220 rpm) at 37 °C in SOC medium [18] supplemented with 3-(*N*-morpholino) propanesulfonic acid (MOPS) sodium salt (pH 8.0, 25 mM) and the respective antibiotics. At an A_588_ of 0.5, IPTG was added to a final concentration of 1 mM. Shaking was continued for 4–5 hours. After centrifugation (4000× *g*, 10 min), cells were resuspended in 30 mM spermidine tris (pH 8), 0,1 M NaCl, and 2 mM EDTA (pH 7.5), frozen in liquid N_2_ and stored at −80 °C until further processing. Preparation of the bacterial cell lysates and all purification steps were performed at 4 °C. Frozen cells were thawed and adjusted to 40 mM Tris-HCl (pH 7.6), 3.5% sucrose, 0.3 mg/mL lysozyme, 0.13% Brij-58 and 1 M NaCl to initiate cell lysis. The cell lysates were incubated on ice for 30 min, frozen in liquid N_2_ and thawed three times to complete lysis. Cell debris was removed from lysates by ultracentrifugation at 70,000× *g* for 90 min. Solid ammonium sulfate [(NH_4_)_2_SO_4_] was added to the supernatant to a saturation of 60% and the preparation was stirred for 30 min [24]. The precipitate was collected by centrifugation at 90,000× *g* for 120 min and dissolved in buffer A (20 mM Tris-HCl (pH 7.6), 1 mM DTT, 0.1 mM EDTA, 10% (*w*/*v*) glycerol) containing 1 M NaCl. Thereafter, the solution was dialyzed three times against buffer A with a stepwise reduction down to a final NaCl concentration of 125 mM (fraction I).

Purification of Cro was achieved by three steps (Table S2). Fraction I containing the resuspended ammonium precipitate in buffer A (125 mM NaCl) was loaded onto a Heparin-Sepharose CL-6B column (2.6 × 15 cm) equilibrated with buffer A containing 125 mM NaCl, and then washed with 250 mL of this buffer. Proteins were eluted with a 500 mL linear gradient of 100–750 mM NaCl in buffer A. Cro was eluted at 450 mM NaCl. Peak fractions were pooled (fraction II). Fraction II was loaded onto a DEAE-Sephacel column (2.6 × 5 cm) equilibrated with buffer A containing 125 mM NaCl. Selected flow-through fractions of Cro were pooled (fraction III). Fraction III was loaded onto a CM-Sepharose column (2.6 × 5 cm) equilibrated with buffer A containing 125 mM NaCl, and then washed with 100 mL of the same buffer. Proteins were eluted with a 500 mL linear gradient of 100–500 mM NaCl in buffer A. Cro eluted at 450 mM NaCl. The peak fraction was concentrated by dialysis against 20% (*w*/*v*) polyethylene glycol (PEG) 20,000 in buffer A and then dialyzed against 50% glycerol in buffer A and stored at −20 °C (fraction IV). Under these conditions, the DNA binding activity of Cro was stable for at least one year.

### 2.5. SDS-PAGE and Mass Spectrometric Analysis

SDS-PAGE was performed according to Laemmli [25]. Samples were suspended in loading buffer, boiled for 10 min, and electrophoresed at 10 V/cm on a 12.5% (*w*/*v*) polyacrylamide (PAA) gel at 15 °C. Proteins were visualized by Coomassie brilliant blue R-250 (Bio-Rad, Munich, Germany) staining. For protein analyses, bands of interest were excised from SDS-gels, digested, and purified as described previously [13,26]. To identify the Cro repressor, high-pressure liquid chromatography (HPLC) coupled to mass spectrometer was used, and automated MS-MS fragmentation was performed during the HPLC run. For the determination of peptide sequences, tandem mass spectrometry (MS-MS) spectra were obtained using a QStar XL hybrid mass spectrometer (Applied Biosystems, Ontario, CA, USA) with a nanoelectrospray source. The obtained data were submitted to the Mascot webserver database (Available online: http://www.matrixscience.com) [27].

### 2.6. Electrophoretic Mobility Shift Assays (EMSA)

In preliminary tests, binding of the PY54 Cro protein to various DNA targets within the *immB*-region was determined by use of recombinant plasmids (Table S1). Target regions of PY54, N15, and ϕKO2 were amplified by PCR from purified phage DNA. Unique restriction sites for BamHI and HindIII were embedded into the sequences of the forward and reverse primers, respectively. Trimmed PCR products were inserted into the corresponding sites of pBR329 [20]. Upon transformation of *E. coli* strain Genehogs, the resulting constructs were verified by sequencing. Relevant information on the plasmids is given in Table S1. After digestion of the constructs with the restriction endonucleases ClaI and HincII, the DNA was incubated with increasing amounts of Cro protein in binding buffer (10 mM Tris-HCl (pH 7.6), 10% glycerol, 0.1 mM EDTA, 0.3 µg/µL BSA, 1 mM DTT) for 30 min at 30 °C. Cro/DNA complexes were analyzed on 1.75% PAA gels in TBE-buffer (89 mM Tris (pH 8.9), 89 mM borate, 1 mM EDTA) at 20 °C. Approximately 100 ng of DNA was incubated with appropriate amounts of purified protein in a final volume of 20 µL. Electrophoresis was carried out under non-denaturing conditions at 6 V/cm for 3.5 h. PAA-gels were stained with SYBR-green (Cambrex BioScience, Rockland, ME, USA) for 30 min and analyzed with the Alpha Digi Doc software (Biometra, Göttingen, Germany).

To determine dissociation constants (K_D_) of Cro binding to different target regions, gel retardation assays with synthetic double-stranded DNA-fragments were performed as described previously [18,28]. 100 ng DNA template were 5′-end labeled with [γ-^32^P] ATP (Hartmann Analytics, Braunschweig, Germany) using T4 polynucleotide kinase (Fermentas, St. Leon-Rot, Germany). After separation of the labeled DNA-fragments from unincorporated nucleotides by MicroSpin G-25 columns (GE Healthcare, Piscataway, PA, USA), a final DNA concentration of 1 nM per reaction was calculated. Binding reactions with the labeled and purified DNA were performed as described above. Protein concentrations are given. Immediately after incubation, samples were applied to 3.5% non-denaturing PAA-gels at 6 V/cm. Gels were vacuum dried prior to autoradiography. Intensities of bands were measured with a PhosphorImager (Molecular Dynamics, Sunnyvale, California, USA) and analyzed with the ImageQuant software (version 3.0, GE Healthcare Life Sciences, Freiburg, Germany). Dissociation constants were determined after fitting the 1:1 binding isotherm to the experimental data by using the SIMFIT program (Available online: http://www.simfit.man.ac.uk).

### 2.7. In Silico Analyses

Sequence analyses and alignments were carried out using the MacVector™ 8.0 software of the Oxford Molecular Group. BLAST searches were performed at the NCBI database [29]. Protein fold analyses and structural comparisons against databases were done by DALI (Available online: http://www.ebi.ac.uk/dali/fssp/), as described by Holm and Sander [30]. For *in silico* promoter studies, the intergenic region between *cI* and *cro* of PY54 was analyzed for the existence of −35 and −10 consensus sequences (TTGACA-N_15-20_-TATAAT) and extended −10 sequences (TGNTATAAT), as described by Hawley and McClure [31], Harley and Reynolds [32] and Kumar *et al.* [33]. The underlined nucleotides of the consensus sequences were specified and up to three mismatches were allowed.

### 2.8. Analysis of Promoter Activity

Promoter activities of the counter-orientated repressor genes *cI* and *cro* were studied using the vector pKKlux (Ap^r^) [34]. pKKlux is a derivative of pKK232-8 that carries the promoterless *luxAB* genes of the bioluminescent bacterium *Vibrio harveyi*. PCR products to be studied for promoter activity were amplified with forward and reverse primers containing restriction sites for SmaI and XbaI, respectively. After digestion, the fragments were inserted into the corresponding sites of pKKlux. Upon transformation of *E. coli* strain Genehogs, the recombinant plasmids pJH281-293 (Table S1) were verified by sequencing. For *in vivo* examinations, the plasmids were introduced into *E. coli* strain DH5α. The luminescence measurements were carried out as previously described [34]. Three independent experiments done in triplicate were conducted and the obtained data were averaged. Cultures of plasmid-containing DH5α strains were grown at 28 °C to an A_600_ of 1.0 and diluted to contain 10^6^ cells per mL. A total volume of 100 µL of the diluted cultures was transferred to a microtiter plate. Fifty microliters of 2% decylaldehyde in 50 mM sodium phosphate buffer (pH 7.0) were added. The luminescence was measured at 28 °C for 10 s in a Microlumat LB96P (EG & G Berthold, Bad Wildbach, Germany). To specify the results of bioluminescence, all constructs were compared with the naked vector pKKlux and pKKL700lux, a plasmid carrying the strong ST-LS1 promoter from *Solanum tuberosum* [34].

### 2.9. Determination of the cI and cro Transcription Start Sites

To determine the transcription start sites of *cI* and *cro*, the intergenic region plus partial sequences of the repressor genes were inserted into the vector pIV2. The construct was introduced into *E. coli* Genehogs. Upon isolation of a positive clone, RNA was isolated from the bacteria using the RNeasy Mini Kit (Qiagen, Hilden, Germany). The 5′-ends of the mRNAs encoding *cI* or *cro* were determined by application of the rapid amplification of cDNA ends (RACE) method using the 5′ RACE System Kit (Invitrogen GmbH, Karlsruhe, Germany) according to the supplier’s protocol. Primers for the determination of the *cro* transcript (5′-GGGAAGGGCGATCGGTGCGGG-3′ and 5′-GGGGATGTGCTGCAAGGCGAT-3′) were binding to vector sequences directly adjacent to the *cro* gene fragment. For *cI*, the primers 5′-CCTTGCCCGTTTTTTTCTAACC-3′ and 5′-CCAATCCCTGTAAGAACCC-3′ with binding sequences within the *cI* gene fragment were used in the RACE protocol. The products were separated on an agarose gel and discrete bands were excised, purified using the QIAquick Gel Extraction kit (Qiagen, Hilden, Germany), and sequenced in an ABI 3730 DNA Analyzer (Applied Biosystems, Foster City, CA, USA).

## 3. Results

### 3.1. cro^PY54^ Triggers PY54 Induced Lysis but Does Not Affect N15 Propagation

Sequence analyses of the genomes of the telomere phages disclosed a similarly organized *immB* region comprising genes potentially coding for a prophage repressor (CI or CB), Cro repressor and antiterminator Q (Figure 2A). The genes encoding the latter two proteins are arranged in one operon [6]. However, while *cro* and *q* are the only cistrons in the N15 and ϕKO2 operon, the PY54 operon contains at least three cistrons in which *cro* and *q* are separated by an open reading frame (ORF42) whose function is unknown. To demonstrate the activity of the PY54 Cro protein under *in vivo* conditions, its gene including potential promoter sequences (see below) was amplified by PCR and inserted into the vector pIV2 which has a copy number of ~14 [22]. The resulting plasmid pJH192 (*cro*^PY54^) and the vector without insert as control were introduced into *E. coli* C-1a and *Y. enterocolitica* 83/88/2. We could not perform experiments with *K. oxytoca* because for ϕKO2, a suitable indicator strain has not been identified. *E. coli* transformants were infected by the N15 mutant D04 containing a *cat* (chloramphenicol acetyltransferase) gene [4], while the corresponding *Yersinia* strains were infected by the PY54 mutant 35Tc, which carries a tetracycline resistance gene (see Materials and Methods). *E. coli* strains containing the vector pIV2 or pJH192 (*cro*^PY54^) were both lysed and lysogenized by N15-D04. On the other hand, pJH192, but not the vector pIV2, prevented lysogenization of *Yersinia* by PY54-35Tc. The data show that the PY54 *cro* gene exclusively acts on PY54 propagation. Since no information has been available about the operator sites of PY54, further experiments focused on the binding properties of its Cro protein.

### 3.2. The Purified cro Product of PY54 Shows Binding to a Specific Target Sequence Within immB

**Figure 3:**
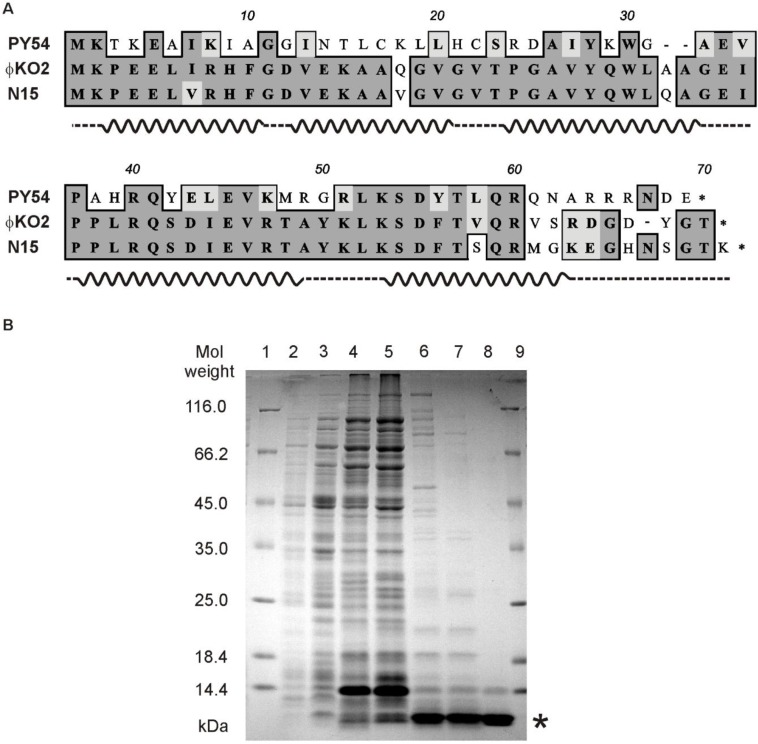
Sequence analysis and purification of PY54 Cro. (**A**) Alignment of the amino acid sequences of the Cro repressors of PY54, ϕKO2 and N15. Similar and identical residues at corresponding positions are shown in light grey and dark grey, respectively. Helices deduced from the structural data on N15 Cro of Dubrava *et al.* [11] are indicated; (**B**) Purification of the PY54 Cro repressor. SDS-PAGE (12.5%) of the crude extract and pooled peak fractions of the three steps of PY54 Cro purification. Lanes 1 and 9, molecular weight marker; lanes 2 and 3, crude extract of the noninduced and IPTG-induced *E. coli* strain SCS1 (pJH142-2) containing the *cro* gene of PY54 (Tables S1 and S2); lane 4, Brij-58/lysozyme extract of induced cells; lane 5, extract after ammonium sulfate precipitation; lane 6, Heparine-Sepharose; lane 7, DEAE-Sephacel; lane 8, CM-Sepharose. The overproduced Cro protein is indicated by an asterisk. Further information on the purification of Cro is given in the Supplemental Material.

To analyze the binding properties on the phage genome by *in vitro* assays, the Cro protein of PY54 was overexpressed in *E. coli*. For that purpose the *cro* structural gene was introduced into the expression vector pMS470Δ8cat containing a *tac* promoter. Following chemical induction, a protein of approximately 8 kDa was produced which is in good agreement with the molecular mass of Cro deduced from the sequence data (Figure 3A). The presence of Cro was confirmed by LC/MS mass spectrometry after excision of the protein band from a SDS polyacrylamide (PAA)-gel and digestion with trypsin [26]. More than 60% of the Cro peptide sequences could be detected by this method.

**Figure 4:**
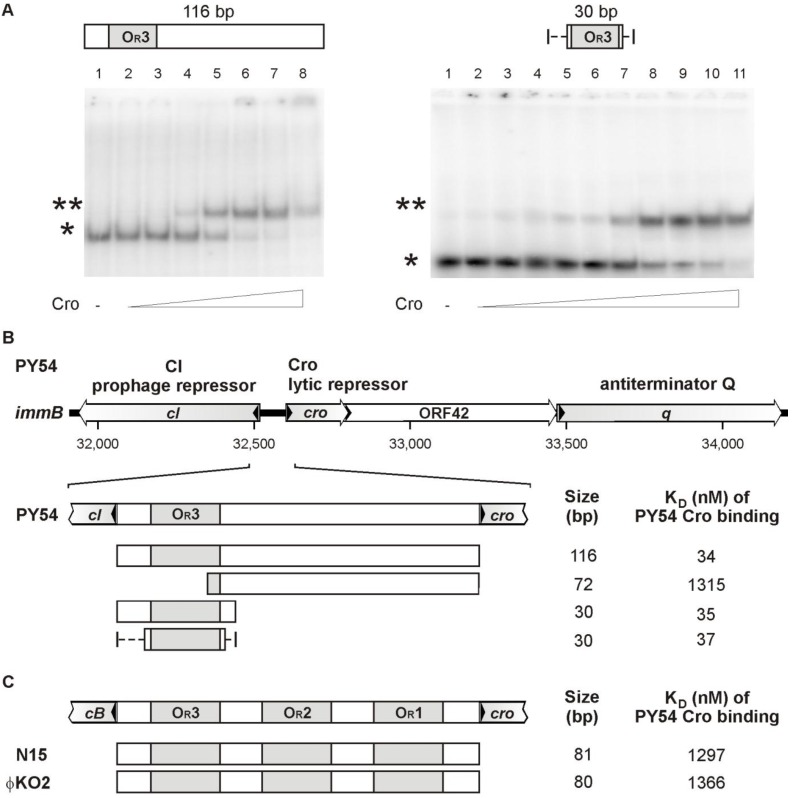
Analysis of the PY54 Cro repressor binding to target DNAs. Binding reactions with ^32^P-5′-labeled oligonucleotides (Table S1) were performed with a final DNA concentration of 1 nM and appropriate amounts of pBR329 as competitor DNA. DNA binding was investigated with various protein concentrations ranging from 0.0 to 0.15 µM. (**A**) In the left gel lanes 1–8 contain the whole intergenic region (116 bp) between the repressor genes *cI* and *cro* incubated with 0, 0.01, 0.025, 0.05, 0.075, 0.1, 0.125 and 0.15 μm of purified Cro protein. In the right gel lanes 1–11 contain a 30 bp target sequence consisting of O_R_3 and nonspecific nucleotides flanking the operator site. Binding to this target was measured with 0, 0.01, 0.02, 0.03, 0.04, 0.05, 0.06, 0.08, 0.1, 0.125 and 0.15 μm purified Cro protein. For each target, the apparent equilibrium dissociation constant K_D_ of PY54 Cro was determined from two independent experiments. The symbols * and ** indicate free and bound DNA, respectively. The depicted line represents artificial nucleotides; (**B**) Localization of the PY54 Cro binding site. The upper part shows a genetic map of PY54 *immB* according to the functional assignment by Hertwig *et al.* [13]. Cro binding assays were performed with ^32^P-5′-labeled oligonucleotides harboring PY54 sequences located between *cI* and *cro* (Table S1). The dotted lines at the ends of the 18 bp palindromic sequence illustrate further random nucleotides required for Cro binding. On the right, the sizes of the respective substrates and the dissociation constants of Cro binding are given; (**C**) Substrates containing the O_R_ operator sites of the phages N15 and ϕKO2.

The repressor was purified close to electrophoretic homogeneity (Figure 3B). Fractions containing Cro were analyzed by *in vitro* binding assays (electrophoretic mobility shift assay, EMSA, see Materials and Methods). The assays were performed with target DNA comprising either cloned PCR products (to identify possible binding sites) or ^32^P-5′-labeled oligonucleotides for quantitative analyses. It was surmised that the intergenic region between the prophage repressor gene and the lytic repressor gene of the phage includes binding sites for Cro because the arrangement of genes within the immunity region is reminiscent of that in lambdoid phages (Figure 2) [35]. Moreover, the N15 prophage repressor CB was already shown to bind to three sites (O_R_, Figure 2B) located between the genes *cB* and *cro* and to two sites (O_L_) located upstream of *repA* [6]. These sites are related in sequence and share bases that are strictly conserved. In ϕKO2, five similar operator sites (three O_R_ and two O_L_ sites) exist, whereas PY54 exhibits only one site (O_R_3) that fits into this scheme (Figure 2B). In a first experiment, binding to the 116, 81 and 80 bp intergenic regions of PY54, N15 and ϕKO2, respectively, was studied. Only the PY54 substrate was shifted in the presence of Cro yielding one DNA-repressor complex (Figure 4A). The measured dissociation constants confirmed that PY54 Cro was specifically bound to the intergenic region of this phage but not to those of N15 and ϕKO2 (Figure 4B,C).

### 3.3. The Cro Repressor Binds to a Single Site Upstream of the Prophage Repressor Gene

To determine the number of Cro binding sites on the PY54 genome, the DNA region between the prophage repressor gene and the *cro* gene was dissected by testing DNA fragments harboring sub-fragments of the intergenic region (Figure 2B). Binding of the PY54 Cro protein exclusively occurred to DNA fragments carrying the O_R_3 site while substrates devoid of this site did not show any binding (Figure 4B). We also tested DNA fragments comprising the upstream region of *repA* (O_L_ operators) and the rest of the genome by use of genomic libraries containing PY54 restriction fragments, which were further digested with other restriction endonucleases to obtain smaller DNA fragments up to 1000 bp. Though, aside from O_R_3, no binding to other PY54 sites was detected. Hence, the Cro repressor of the telomere phage PY54 binds to a single site on the phage genome located immediately upstream of the prophage repressor gene. The *in vitro* assays reinforced the *in vivo* findings in such a way that the PY54 Cro repressor specifically recognized the PY54 O_R_3 site but not the closely related O_R_3 sites of N15 and ϕKO2 (Figure 4C).

### 3.4. The Central 16 bp of O_R_3 Are the Target of Cro

**Figure 5:**
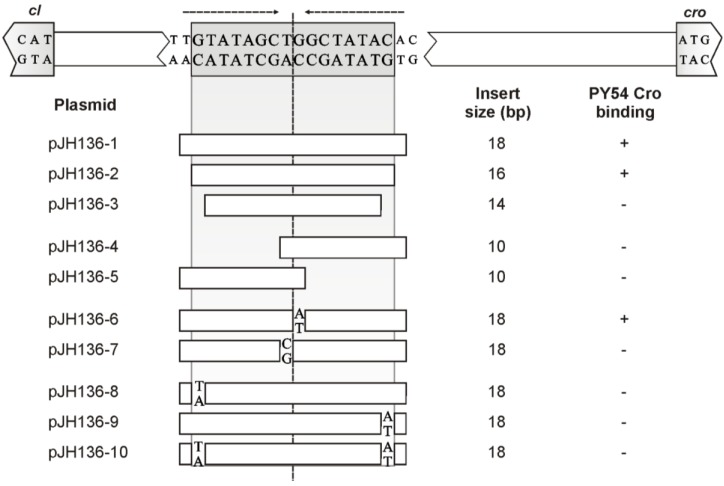
Sequence requirements and mutational analysis of the PY54 Cro binding site. At the top the nucleotide sequence of the upstream region of the prophage repressor gene *cI* is shown. The O_R_3 site exhibiting two-fold rotational symmetry is boxed. Arrows represent nucleotides with two-fold rotational symmetry. The lower part lists the substrates used for binding assays. Base pair exchanges in mutants are indicated. On the right, the sizes of the inserts and the results of the Cro binding tests are given (+, binding; −, no binding). This set of experiments was performed with substrates comprising annealed oligonucleotides inserted in pBR329 (see Materials and Methods) because these short target sequences were specifically bound only in the presence of additional (random) bases flanking the oligonucleotides.

Previous experiments showed a 30 bp fragment harboring the O_R_3 site of PY54 to be sufficient for Cro binding (Figure 4A). To define the actual target of Cro, the size of the constructs was further reduced. The O_R_3 site of PY54 comprises 18 bp possessing two-fold rotational symmetry (Figure 2B). Quantitative experiments with the respective ^32^P-5′-labeled 18 bp oligonucleotides showed no binding of Cro (data not shown). However, specific binding was achieved when O_R_3 was flanked by additional (random) bases (Figure 4B) or by use of fragments of substrate plasmids harboring O_R_3 or O_R_3 derivatives. However, half sites of O_R_3 were not bound by Cro (Figure 5). In contrast to PY54 and ϕKO2, the O_R_3 site of N15 consists of only 16 bp exhibiting two-fold rotational symmetry (Figure 2B). This sequence is identically present in ϕKO2 and was already shown to be the target for the N15 prophage repressor CB [6]. Therefore, we also studied binding of the PY54 Cro repressor to the inner 16 bp of O_R_3 and found that this short stretch is indeed sufficient for Cro binding. Thus, the target recognized by the PY54 Cro protein could be narrowed down to the central 16 bp within O_R_3. Comparison of the O_R_3 sites revealed identity between N15 and ϕKO2, whereas the PY54 operator sequence diverges by its peripheral base pairs. From the binding studies, it can be reasoned that the terminal nucleotides of the operator sites are important for the recognition by the Cro proteins of the telomere phages.

### 3.5. Both Peripheral Base Pairs of O_R_3 Determine Phage Specificity

The first (G) and the last (C) nucleotide of the PY54 O_R_3 site deviate from the O_R_3 sites of the other two phages (Figure 2B). Since the outermost nucleotides are essential for binding, they determine Cro specificity. After replacement of one terminal nucleotide, e.g., G with T or C with A within the PY54 sequence, binding of the PY54 Cro repressor was abrogated (Figure 5). We also studied the importance of the central T-G nucleotides within the PY54 O_R_3 sequence. Constructs containing an A instead of the G were still bound by Cro, whereas replacement of the T by a C resulted in a loss of binding (Figure 5). Therefore, a fully symmetrical O_R_3 site is also recognized by PY54 Cro if the center is occupied by a T-A motif […GCT-AGC…]. On the other hand, the symmetrical sequence [...GCC-GGC...] does not support binding, probably because of significant conformational alterations induced by this GC tract.

### 3.6. Promoters of the Repressor Genes Center on the cro Region

**Figure 6:**
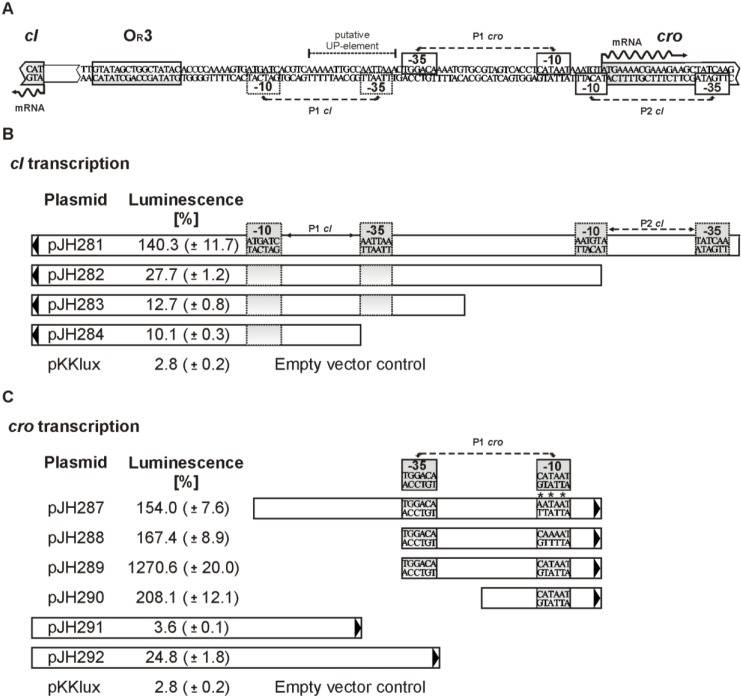
Identification of functional *cI* and *cro* promoters in PY54. (**A**) Sequence of the DNA region between the repressor genes *cI* and *cro*. The Cro binding site and promoter sequences identified by luciferase assays are boxed; (**B**) DNA fragments analyzed for *cI* promoter activity. Arrows indicate the direction in which the fragments were inserted into the promoter search vector pKKlux (Table S1). Transcription activities (bioluminescence) of the constructs compared to those of pKKL700 (ST-LS1 promoter = 100%) are given. Standard deviations are shown in brackets; (**C**) Constructs tested for *cro* promoter activity. Mutations found within the −10 region [CATAAT] are depicted by asterisks. The encircled nucleotide A in pJH287 and pJH288 indicates the replacement of the nucleotides C and T, respectively.

The arrangement of *cI* (*cB*) and *cro* on the genomes of the telomere phages suggests the presence of at least two divergent promoters located within the intergenic region between the oppositely situated genes (Figure 2A). On N15, several possible promoters for *cB* and *cro* have been predicted [6]. Similarly, promoter sequences can be found in ϕKO2. To determine the actual positions and activities of *cI* promoters in PY54, we introduced the intergenic region, including adjacent sequences of the repressor genes and sub-fragments of the region into the promoter search vector pKKlux [34]. Transcription activities of constructs were measured by luminescence and compared with the control plasmids pKKlux and pKKL700lux (see Materials and Methods). The *cI* promoter P1 (plasmid pJH283) of PY54, which is easily detectable by *in silico* analysis revealed only weak activity (Figure 6B). A much stronger *cI* promoter (P2, plasmid pJH281) was found at the 5′-end of *cro*. Even a construct (pJH282) lacking the −35 region of this promoter gave significant transcription activity. Plasmid pJH284 harboring the left half of the intergenic region exhibited transcription activity only slightly higher than the negative control (pKKlux). The data on PY54 indicate that *cI* expression is under control of two promoters, of which the upstream promoter possesses a more than 10-fold higher activity.

Attempts were also made to identify active *cro* promoters on the PY54 genome. Surprisingly, we initially failed to obtain plasmids containing unchanged DNA fragments in front of *luxAB*, which harbored the region efficiently bound by the RNA polymerase. All inserts of the analyzed plasmids exhibited point mutations or T deletions, most of which resided within the short sequence [...CATAAT...], a suitable -10 promoter region. Out of 14 mutations detected on the inserts (64 bp and 45 bp) of the plasmids pJH287 and pJH288, three mutations affected nucleotide C, while the middle T and peripheral T of this promoter sequence were affected by five and six mutations, respectively (Figure 6C). The same PY54-DNA could be amplified without generating mutations by molecular cloning of the fragments in the opposite direction. Furthermore, for Cro binding studies, each of the DNA-fragments including the complete intergenic region of the phages was amplified by help of pBR329 and did not disclose any mutations. Thus, we concluded that in PY54 the DNA-region upstream of *cro* contains a strong promoter whose activity probably leads to an overexpression of *luxAB,* the product of which likely inhibits cell growth when produced in higher amounts. This assumption was corroborated by the fact that the constructs harboring mutations still exhibited high transcription activities (Figure 6C). To avoid toxic effects, transformants harboring the putative PY54 *cro* promoter (pJH289) were selected on M9 minimal medium [18]. In this way a strain was isolated containing the unchanged *cro* promoter sequences. Plasmid pJH289 gave by far the highest activity of all tested constructs. Even its derivative pJH290 only containing the -10 region of the PY54 *cro* promoter revealed high activity (Figure 6C).

We also determined the transcription start sites of the mRNAs encoding CI and Cro by sequencing of RACE products. The experiment was carried out using a DNA fragment, which contained the intergenic region and partial sequences of the repressor genes (see Material and Methods). For each repressor, only one species of a leaderless mRNA was identified beginning with the AUG start codon of *cI* or *cro* (Figure 6A).

### 3.7. The C-Terminal Region of Cro is Essential for O_R_3 Binding

**Figure 7:**
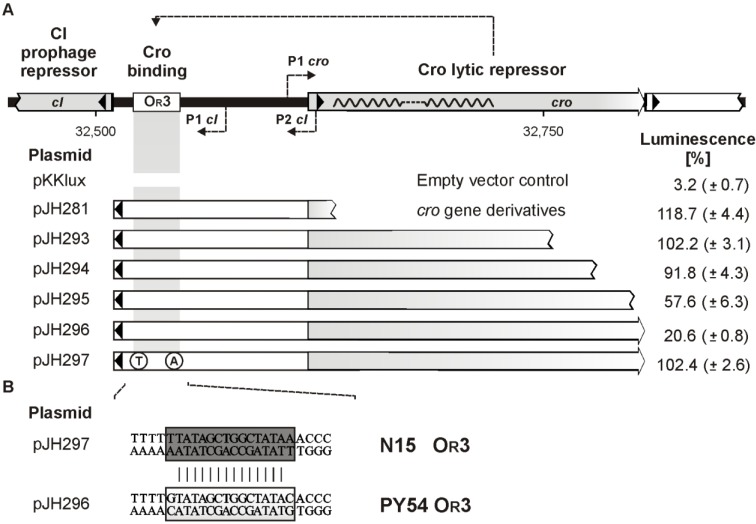
Repression of PY54 *cI* transcription by Cro binding to O_R_3. (**A**) At the top, the repressor genes, the O_R_3 operator site and the identified *cI* and *cro* promoters within the intergenic region of PY54, are shown. Constructs tested for bioluminescence activity are listed below. Black arrowheads indicate the direction (towards *cI*) in which the fragments were inserted into the promoter search vector pKKlux. Spontaneous mutants exhibiting base pair exchanges within O_R_3 are also presented. On the right, transcription activities of the constructs compared to those of the naked vector pKKlux and plasmid pKKL700 (ST-LS1 promoter = 100 %) are given. Standard deviations are shown in brackets; (**B**) Alignment of the PY54 and N15 O_R_3 operator sequences.

To study the influence of Cro binding on the transcription of the prophage repressor gene in detail, luciferase assays were performed with constructs comprising the intergenic region and either the complete or truncated *cro* gene. Expression of all constructs was verified by SDS-PAGE (data not shown). Plasmid pJH296 that contained the whole PY54 *cro* gene showed a very low luminescence indicating that Cro binding to its target O_R_3 almost completely blocked the transcription of *luxAB* (Figure 7A). Mutations within O_R_3 resulted in higher luminescence values probably due to decreased binding of Cro. Most notably, the mutation in plasmid pJH297 creating the N15/ϕKO2 O_R_3 site gave nearly the same high luminescence like constructs containing a defective *cro* gene (e.g., pJH293). This result affirmed that the N15/ϕKO2 O_R_3 site is not a target of the PY54 Cro repressor.

Constructs in which the 3′-end of *cro* was truncated by 9 bp (pJH295) and 27 bp (pJH294), gave approximately threefold and four-and-a-half-fold higher luminescence values, respectively, as the complete *cro* gene (Figure 7A). Further truncation of *cro* resulted in an only slight increase of luminescence. The data indicate that C-terminal amino acids of the PY54 Cro repressor are essential for transcriptional repression. The C-terminus of Cro may be important for the recognition and binding of the target, for the folding of the protein, for its stability, or for the dimerization of the lytic repressor.

## 4. Discussion

Phage λ and many lambda-like phages posses a similarly organized immunity region essential for their regulatory circuitries [35]. The region contains the repressor genes *cI* and *cro*, *N* and *cII* encoding an antiterminator and transcriptional activator, respectively, and *cIII*, which is important for the stabilization of CII. In addition, the region harbors two sites (O_R_, O_L_) containing six operators, the targets of the repressor proteins. Contrary to the situation in λ, the regulatory genes of the telomere phages are arranged in several loci. In N15 the immunity regions *immA* and *immB* and the separate repressor gene *antC* have been identified [5]. A similar organization apparently exists in ϕKO2, while PY54 contains a locus related to *immB* and some individual genes (*antA*, *icd*) scattered on the phage genome [1,13].

Studies that have been performed on the immunity region *immB* of N15 suggested that the interplay between CB and Cro in the telomere phages resembles those described for λ [6]. The genes for the repressors are divergently arranged and three (one in PY54) easily identifiable operator sites (O_R_) are lying in between. Indeed, DNase I footprints of the N15 CB repressor indicated binding to the O_R_ operators, which are overlapping with predicted promoters of *cro* and *cB*. In λ, Cro binds to the same operators as CI but non-cooperatively and with reversed affinity [36]. Analyses of Cro recognition helices and O_R_ half-site sequences of lambdoid bacteriophages (amongst others, the telomere phage N15) suggested common rules of recognition [12]. Due to the strong sequence homologies of the ϕKO2 and N15 Cro proteins, and also of the O_R_ sites of the phages (Figure 2 and Figure 3), it is likely that Cro binding in these two phages occurs similarly. Though, our studies indicate that the genetic switch of PY54 diverges significantly from those of many other lambda-like phages. In PY54 binding of the lytic repressor is restricted to the O_R_3 site upstream of the prophage repressor gene. Other sites on the phage genome could not be identified, neither by *in silico* analyses, nor by experimental approaches. While λ Cro’s affinities for O_R_2 and O_R_1 could be clearly demonstrated and quantified (about tenfold lower than that for O_R_3), the dissociation constants measured in this study did not give any clue for PY54 Cro binding besides to O_R_3. The PY54 Cro repressor is very specific in terms of recognition and binding to its targets as it does not bind to the nearly identical ϕKO2 and N15 O_R_3 operator site. We show that phage specificity in fact can be attributed to the two peripheral base pairs of O_R_3, the only nucleotides different in the operators of PY54 and ϕKO2/N15.

**Figure 8:**
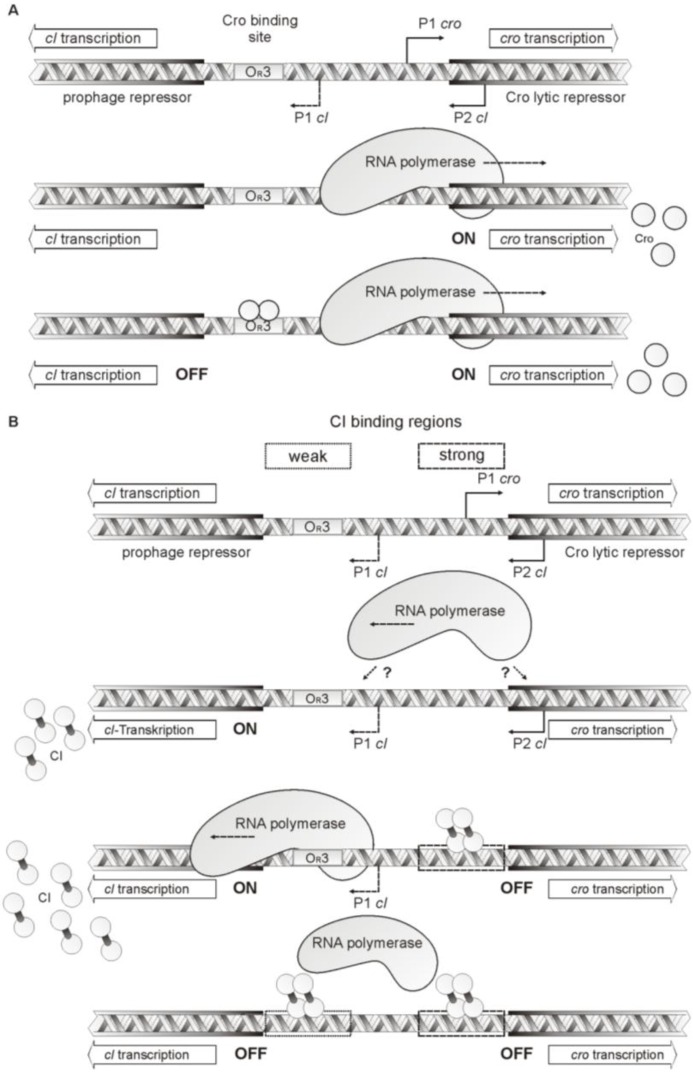
Model for PY54 repressor activity. (**A**) Influence of PY54 lytic repressor binding on *cI* and *cro* transcription. The upper part shows the location of *cI* and *cro*, the positions of the identified promoters and the Cro binding site O_R_3. In the middle, binding of the RNA polymerase to the strong *cro* promoter is illustrated. This leads to *cro* transcription and the synthesis of lytic repressor molecules. Whether there is any transcription of *cI* at this stage, is unknown. Upon Cro synthesis, the protein binds to O_R_3 resulting in a blocking of *cI* transcription whereas transcription of *cro* can still take place; (**B**) Influence of PY54 prophage repressor binding on *cI* and *cro* transcription. The upper part shows the location of *cI* and *cro*, the positions of the identified promoters, the Cro binding site and CI binding regions. In the next diagram, possible binding of the RNA polymerase to the *cI* promoters is illustrated. It leads to *cI* transcription and the synthesis of prophage repressor molecules. Whether there is any transcription of *cro* at this stage, is unknown. Upon CI synthesis, the protein binds next to the start of the *cro* gene resulting in a blocking of *cro* transcription, whereas transcription of *cI* can still take place. High concentrations of CI molecules may lead to additional binding next to *cI* resulting in the repression of *cI* transcription.

What does binding of Cro to a single site mean? In PY54, Cro down-regulates repressor synthesis by blocking transcription of the repressor gene, as demonstrated by our luciferase assays. However, unlike lambda, Cro is apparently not able to repress its own expression when present in high concentration, because binding to the O_R_3 site alone may not interfere with the transcription of *cro* (Figure 8A). We identified strong promoter sequences immediately upstream of the *cro* start codon correlating with the determined transcription initiation site of a leaderless mRNA for *cro* expression. As this promoter is up to 40 bp apart from O_R_3, the distance might be too long for a direct interaction of Cro with the RNA polymerase (Figure 6 and Figure 8A). An adenine and thymine-rich sequence similar to an UP element is located adjacent to the *cro* promoter, which might interact with the C-terminal domain of the RNAP α-subunit (Figure 6) [37]. Since it is yet not clear whether binding of the α-subunit to this site occurs, and whether it can be influenced by Cro, it remains open whether a negative autoregulation of Cro concentration exists in PY54.

Another interesting aspect pertains to the regulation of *repA*. The N15 prophage repressor was reported to bind not only to the O_R_ operators but also to two O_L_ sites located upstream of *repA* [6]. In this way, CB regulates the copy number of the plasmid prophage. By contrast, the PY54 Cro protein was not bound to the O_L_ sites indicating that at least this repressor is not implicated in plasmid replication. Moreover, the complete genome of PY54 only contains a single copy of the O_R_3 operator site.

N15 Cro which shows 25% and 9% identity to the Cro proteins of P22 and lambda, respectively, has been subjected to X-ray diffraction [11]. It exhibited two chains both of which containing five α-helices. By its structure, N15 Cro much more resembles the all-α fold class representative P22 Cro than lambda Cro, the paradigm of the α + β class. However, dimerization of the N15 lytic repressor occurred in solution like lambda Cro, much stronger than of its structural homolog in P22. For that reason, N15 Cro was suggested to represent a new category of Cro proteins, the all-α helical dimer to which most likely the ϕKO2 lytic repressor belongs as well [11]. In the same study, PY54 Cro was also briefly characterized. This protein, which is 33% identical in sequence to N15 Cro over 60 residues, dimerized at least as strong as its N15 counterpart. Thus, PY54 Cro might be a member of the all-α helical dimer group, too. It is conceivable that Cro proteins belonging to this new class exhibit unusual properties, e.g., an extraordinary high specificity for their target sequences. In spite of the fact that unlike PY54, N15 and ϕKO2 contain several similar operator sites, binding of their Cro repressors might be restricted to O_R_3 because the three operator sequences of N15 and ϕKO2 deviate more from each other than from the closely related PY54 O_R_3 site. The predicted structure of N15 Cro suggests that each half-site of the palindromic operator DNA binds one copy of the protein in the major groove, similar to the proteins mentioned above. Though, crystal structures of N15 Cro have been studied without its target DNA. For lambda Cro, different structures have been obtained depending on the presence of operator sequences [38]. Hence, to describe the binding mode, structure analyses with Cro should be conducted on Cro/DNA complexes. Preliminary *in vivo* studies on the N15 and ϕKO2 Cro activity revealed no inhibition of PY54 lysogenization, indicating that these repressors are, like their PY54 counterpart, highly specific with respect to their target (data not shown). This speculation should be verified by determination of the real N15 and ϕKO2 Cro operator sites.

Preliminary studies on the PY54 CI repressor indicated that this protein specifically binds to two sites within the intergenic region (to be published elsewhere). Strong binding was observed to a region covering the *cro* promoter while a weaker affinity was found for a region encompassing O_R_3 (Figure 8B). Thus, the PY54 CI repressor binds to sites that share only marginal sequence similarity. This is another striking difference to the lambda and N15 molecular switch. As with Cro, the PY54 CI sequence diverges significantly from the repressors of N15, ϕKO2 and lambda (Figure S1), which may explain its unusual binding specificity. 

By which promoter *cI* expression is regulated in PY54 is not clear and has still to be elucidated. Similar to the lambda prophage repressor gene [39], *cI* of PY54 is transcribed as leaderless mRNA, which means that a RNA Polymerase molecule bound to the P2 promoter would have to slide a rather long distance to reach the *cI* transcription start site (Figure 8). Moreover, binding of the prophage repressor to the primary binding region upstream of *cro* does not only block Cro synthesis but presumably also represses its own transcription from the P2 promoter. CI transcription may of course also be regulated by the much weaker promoter P1 and may be enhanced by activation of the RNA polymerase. In lambda, CI is not only a negative, but also a positive regulator, which increases its own expression by interacting with the RNA polymerase. Similarly, the PY54 CI repressor might repress its own synthesis at high concentrations by binding to the O_R_3 region.

The PY54 *immB* region contains a gene (ORF42) whose function is not yet known. Interestingly, proteins homologous to the ORF42 gene product also occur in marine telomere phages (Vp58.5, VP882, vB_VpaM_MAR, phiHAP-1), but not in N15 and ϕKO2. As in PY54, the respective gene is located within the immunity region of the phages [13,19,40,41,42,43]. While *cI* repressor genes have been predicted in all marine telomere phages, *cro*-like genes have not been identified. Instead, the ORF42-related gene is located at the position of *cro* in these phages. This raises the question, whether PY54 ORF42 or its relatives may act as repressors. For this reason, we studied ORF42 activity under *in vivo* and *in vitro* conditions, but did neither detect any influence on phage lifestyle nor any binding to PY54 DNA (data not shown). Therefore, it remains open which role this gene plays.

In conclusion, our studies demonstrated that, compared to lambda and N15/ϕKO2, the molecular switch of PY54 shows some striking differences. A view to the marine telomere phages ϕHAP-1, VP58.5 and VP882 reveals that the molecular switch of these phages might be even more diverse since their intergenic regions between *cI* and *cro* do not contain any putative operator site readily detectable from the DNA sequences. Combined with the fact that these phages possess an ORF42-related protein, encoded within the immunity region, it appears that the PY54 molecular switch is halfway between the molecular switches of N15/ϕKO2 and marine telomere phages.

## Supplementary Materials

Supplementary materials can be found at http://www.mdpi.com/1999-4915/7/6/2771/s1.

## References

[1] Casjens S.R., Gilcrease E.B., Huang W.M., Bunny K.L., Pedulla M.L., Ford M.E., Houtz J.M., Hatfull G.F., Hendrix R.W. (2004). The pKO2 linear plasmid prophage of *Klebsiella oxytoca*. J. Bacteriol..

[2] Hertwig S., Klein I., Lurz R., Lanka E., Appel B. (2003). PY54, a linear plasmid prophage of *Yersinia enterocolitica* with covalently closed ends. Mol. Microbiol..

[3] Rybchin V.N., Svarchevsky A.N. (1999). The plasmid prophage N15: A linear DNA with covalently closed ends. Mol. Microbiol..

[4] Hammerl J.A., Klein I., Appel B., Hertwig S. (2007). Interplay between the temperate phages PY54 and N15, linear plasmid prophages with covalently closed ends. J. Bacteriol..

[5] Mardanov A.V., Ravin N.V. (2007). The antirepressor needed for induction of linear plasmid-prophage N15 belongs to the SOS regulon. J. Bacteriol..

[6] Lobocka M.B., Svarchevsky A.N., Rybchin V.N., Yarmolinsky M.B. (1996). Characterization of the primary immunity region of the *Escherichia coli* linear plasmid prophage N15. J. Bacteriol..

[7] Ravin N.V., Svarchevsky A.N., Deho G. (1999). The anti-immunity system of phage-plasmid N15: Identification of the antirepressor gene and its control by a small processed RNA. Mol. Microbiol..

[8] Hochschild A., Douhan J., Ptashne M. (1986). How lambda repressor and lambda Cro distinguish between OR1 and OR3. Cell.

[9] Ziegelin G., Tegtmeyer N., Lurz R., Hertwig S., Hammerl J.A., Appel B., Lanka E. (2005). The *repA* gene of the linear *Yersinia enterocolitica* prophage PY54 functions as a circular minimal replicon in *Escherichia coli*. J. Bacteriol..

[10] Ravin N.V. (2011). N15: The linear phage-plasmid. Plasmid.

[11] Dubrava M.S., Ingram W.M., Roberts S.A., Weichsel A., Montfort W.R., Cordes M.H. (2008). N15 Cro and lambda Cro: orthologous DNA-binding domains with completely different but equally effective homodimer interfaces. Protein Sci..

[12] Hall B.M., Lefevre K.R., Cordes M.H. (2005). Sequence correlations between Cro recognition helices and cognate O(R) consensus half-sites suggest conserved rules of protein-DNA recognition. J. Mol. Biol..

[13] Hertwig S., Klein I., Schmidt V., Beck S., Hammerl J.A., Appel B. (2003). Sequence analysis of the genome of the temperate *Yersinia enterocolitica* phage PY54. J. Mol. Biol..

[14] Ravin V., Ravin N., Casjens S., Ford M.E., Hatfull G.F., Hendrix R.W. (2000). Genomic sequence and analysis of the atypical temperate bacteriophage N15. J. Mol. Biol..

[15] Hammerl J.A., Klein I., Lanka E., Appel B., Hertwig S. (2008). Genetic and functional properties of the self-transmissible *Yersinia enterocolitica* plasmid pYE854, which mobilizes the virulence plasmid pYV. J. Bacteriol..

[16] Hammerl J.A., Freytag B., Lanka E., Appel B., Hertwig S. (2012). The pYV virulence plasmids of *Yersinia pseudotuberculosis* and *Y. pestis* contain a conserved DNA region responsible for the mobilization by the self-transmissible plasmid pYE854. Environ. Microbiol. Rep..

[17] Hertwig S., Klein I., Hammerl J.A., Appel B. (2003). Characterization of two conjugative *Yersinia* plasmids mobilizing pYV. Adv. Exp. Med. Biol..

[18] Sambrook J., Russel D. (2001). Molecular Cloning: A Laboratory Manual.

[19] Hertwig S., Klein I., Appel B. (2003). Properties of the temperate *Yersinia enterocolitica* bacteriophage PY54. Adv. Exp. Med. Biol..

[20] Covarrubias L., Bolivar F. (1982). Construction and characterization of new cloning vehicles. VI. Plasmid pBR329, a new derivative of pBR328 lacking the 482-base-pair inverted duplication. Gene.

[21] Schiemann D.A. (1979). Synthesis of a selective agar medium for *Yersinia enterocolitica*. Can. J. Microbiol..

[22] Strauch E., Voigt I., Broll H., Appel B. (2000). Use of a plasmid of a *Yersinia enterocolitica* biogroup 1A strain for the construction of cloning vectors. J. Biotechnol..

[23] Balzer D., Ziegelin G., Pansegrau W., Kruft V., Lanka E. (1992). KorB protein of promiscuous plasmid RP4 recognizes inverted sequence repetitions in regions essential for conjugative plasmid transfer. Nucl. Acids Res..

[24] Di Jeso F. (1968). Ammonium sulfate concentration conversion nomograph for 0 degrees. J. Biol. Chem..

[25] Laemmli U.K. (1970). Cleavage of structural proteins during the assembly of the head of bacteriophage T4. Nature.

[26] Rosenfeld J., Capdevielle J., Guillemot J.C., Ferrara P. (1992). In-gel digestion of proteins for internal sequence analysis after one- or two-dimensional gel electrophoresis. Anal. Biochem..

[27] Perkins D.N., Pappin D.J., Creasy D.M., Cottrell J.S. (1999). Probability-based protein identification by searching sequence databases using mass spectrometry data. Electrophoresis.

[28] Hellman L.M., Fried M.G. (2007). Electrophoretic mobility shift assay (EMSA) for detecting protein-nucleic acid interactions. Nat. Protoc..

[29] Altschul S.F., Madden T.L., Schaffer A.A., Zhang J., Zhang Z., Miller W., Lipman D.J. (1997). Gapped BLAST and PSI-BLAST: A new generation of protein database search programs. Nucl. Acids Res..

[30] Holm L., Sander C. (1993). Protein structure comparison by alignment of distance matrices. J. Mol. Biol..

[31] Hawley D.K., McClure W.R. (1983). Compilation and analysis of *Escherichia coli* promoter DNA sequences. Nucl. Acids Res..

[32] Harley C.B., Reynolds R.P. (1987). Analysis of *E. coli* promoter sequences. Nucl. Acids Res..

[33] Kumar A., Malloch R.A., Fujita N., Smillie D.A., Ishihama A., Hayward R.S. (1993). The minus 35-recognition region of *Escherichia coli* sigma 70 is inessential for initiation of transcription at an “extended minus 10” promoter. J. Mol. Biol..

[34] Jacob D., Lewin A., Meister B., Appel B. (2002). Plant-specific promoter sequences carry elements that are recognised by the eubacterial transcription machinery. Transgenic Res..

[35] Degnan P.H., Michalowski C.B., Babic A.C., Cordes M.H., Little J.W. (2007). Conservation and diversity in the immunity regions of wild phages with the immunity specificity of phage lambda. Mol. Microbiol..

[36] Johnson A., Meyer B.J., Ptashne M. (1978). Mechanism of action of the cro protein of bacteriophage lambda. Proc. Natl. Acad. Sci. USA.

[37] Estrem S.T., Gaal T., Ross W., Gourse R.L. (1998). Identification of an UP element consensus sequence for bacterial promoters. Proc. Natl. Acad. Sci. USA.

[38] Hall B.M., Roberts S.A., Heroux A., Cordes M.H. (2008). Two structures of a lambda Cro variant highlight dimer flexibility but disfavor major dimer distortions upon specific binding of cognate DNA. J. Mol. Biol..

[39] Resch A., Tedin K., Graschopf A., Haggard-Ljungquist E., Blasi U. (1995). Ternary complex formation on leaderless phage mRNA. FEMS Microb. Rev..

[40] Alanis V.A., Kropinski A.M., Abbasifar R., Griffiths M.W. (2012). Complete genome sequence of *Vibrio parahaemolyticus* bacteriophage vB_VpaM_MAR. J. Virol..

[41] Lan S.F., Huang C.H., Chang C.H., Liao W.C., Lin I.H., Jian W.N., Wu Y.G., Chen S.Y., Wong H.C. (2009). Characterization of a new plasmid-like prophage in a pandemic *Vibrio parahaemolyticus* O3:K6 strain. Appl. Environ. Microb..

[42] Mobberley J.M., Authement R.N., Segall A.M., Paul J.H. (2008). The temperate marine phage PhiHAP-1 of *Halomonas aquamarina* possesses a linear plasmid-like prophage genome. J. Virol..

[43] Zabala B., Hammerl J.A., Espejo R.T., Hertwig S. (2009). The linear plasmid prophage Vp58.5 of *Vibrio parahaemolyticus* is closely related to the integrating phage VHML and constitutes a new incompatibility group of telomere phages. J. Virol..

