# Supplementary material for: The Molecular Switch of Telomere Phages: High Binding Specificity of the PY54 Cro Lytic Repressor to a Single Operator Site
Source: Viruses. 2015 Jun 2;7(6):2771–93. doi: 10.3390/v7062746 (PMC4488713; doi:10.3390/v7062746)
Supplement: Supplementary file 1 [file viruses-07-02746-s001.pdf]

Supplemental Information

Table S1. Bacterial strains, phages and plasmids.

Strains	Description	Reference or origin
E. coli SCS1	*recA1 endA1 gyrA*96 *thi-1 hsdR17*(rK-mK+) *supE44 relA1*	Stratagene
E. coli UT5600	F- *ara-14 leuB6 secA6 lacY1 proC14 tsx-67* Δ(*ompT-fepC*)266 *entA403 trpE38 rfbD1 rpsL109 xyl-5 mtl-1 thi-1*	NEB
E. coli Genehogs	F- *mcrA* Δ(*mrr-hsdRMS-mcrBC*) ϕ80 *lacZ* ΔM15 Δ*lacX74 recA1 araD139* Δ(*ara-leu*)7697 *galU galK rpsL* (Smr) *endA1 nupG fhuA*::IS2 (confers phage T1 resistance)	Invitrogen
E. coli C-1a	derived from C-1; fertility type F-, prototrophic	[1]
E. coli C-1a (N15)	derived from C-1; fertility type F-, prototrophic; contains the N15 prophage	G. Deho
Y. enterocolitica 83/88/2	serogroup O:5,27; biogroup 2 strain; isolated from man; cured from *Yersinia* virulence plasmid (pYV)	[2–4]
Y. enterocolitica 29854	serogroup O:5; biogroup 1A strain; isolated from food; contains the PY54 prophage	[2–4]
K. oxytoca CCUG 15788	wild type strain, isolated from a mineral oil emulsion tank in Göteborg, Sweden; contains the ϕKO2 prophage	[5]
**Phages**	**Description**	**Reference or Origin**
PY54	46.3 kb; isolated from *Y. enterocolitica* strain 29854	[2–4]
PY54-35Tc	PY54 mutant; Tcr inserted into ORF35	This work
N15	46.3 kb; isolated from sewage	[6–8]
N15-D04	N15 mutant; Cmr inserted into ORF36	[9]
ϕKO2	51.6 kb, isolated from *K. oxytoca* strain CCUG 15788	[5]
**Plasmids**	**Description**	**Reference or Origin**
pBR329	4.2 kb cloning vector, pMB1 replicon; Apr, Cmr, Tcr	[10]
pMS470Δ8*cat*	6.9 kb cloning vector, pMB1 replicon, P*tac*/ *lacI*; T7 gene Shine-Dalgarno sequence; Apr, Cmr	[11]
pIV2	3.6 kb *Yersinia* cloning vector; Kmr	[12]
pLitmus38	2.8 kb cloning vector; Apr	NEB
pKKlux	7.7 kb promoter probe vector; pMB1 replicon; promoterless *luxAB* genes from *Vibrio harveyi*; T1T2 of *rrnB*; Apr	[13]
pKKL700lux	9.3 kb; derived from pKKlux, contains the *Solanum tuberosum* ST-LS promoter; Apr	[13]

Table S1. *Cont.*

Plasmids	Description	Reference or Origin
pJH136-1	contains the 18 bp palindromic OR3 operator sequence	Figure 5, this work
	pBR329 Δ[BamHI-HindIII] Ω[PCR, 18 bp, BamHI-HindIII], PY54 32,535–32,552	
pJH136-2	contains the 16 bp OR3 operator sequence	Figure 5, this work
	pBR329 Δ[BamHI-HindIII] Ω[PCR, 16 bp, BamHI-HindIII], PY54 32,536–32,551	
pJH136-3	contains 14 bp of the central OR3 operator	Figure 5, this work
	pBR329 Δ[BamHI-HindIII] Ω[PCR, 14 bp, BamHI-HindIII], PY54 32,537–32,550	
pJH136-4	contains the right-arm of the OR3 palindrome	Figure 5, this work
	pBR329 Δ[BamHI-HindIII] Ω[PCR, 10 bp, BamHI-HindIII], PY54 32,543–32,552	
pJH136-5	contains the left-arm of the OR3 palindrome	Figure 5, this work
	pBR329 Δ[BamHI-HindIII] Ω[PCR, 10 bp, BamHI-HindIII], PY54 32,535–32,544	
pJH136-6	contains the 18 bp palindromic OR3 operator with a central A/T nucleotide change	Figure 5, this work
	pBR329 D[BamHI-HindIII] W[PCR, 18 bp, BamHI-HindIII], PY54 32,535–32,552	
pJH136-7	contains the 18 bp palindromic OR3 operator with a central G/C nucleotide change	Figure 5, this work
	pBR329 D[BamHI-HindIII] W[PCR, 18 bp, BamHI-HindIII], PY54 32,535-32,552	
pJH136-8	contains the 18 bp palindromic OR3 operator with a partial adaption to the N15 OR3 operator	Figure 5, this work
	pBR329 D[BamHI-HindIII] W[PCR, 18 bp, BamHI-HindIII], PY54 32,535–32,552	
pJH136-9	contains the 18 bp palindromic OR3 operator with a partial adaption to the N15 OR3 operator	Figure 5, this work
	pBR329 D[BamHI-HindIII] W[PCR, 18 bp, BamHI-HindIII], PY54 32,535–32,552	
pJH136-10	contains the 18 bp palindromic OR3 operator of N15	Figure 5, this work
	pBR329 D[BamHI-HindIII] W[PCR, 18 bp, BamHI-HindIII], PY54 32,535–32,552	
pJH142-2	PY54 *cro* gene expression plasmid	This work
	pMS470D8*cat* D[NdeI-HindIII] W[PCR, *cro* gene, NdeI-HindIII], PY54 32,629–32,831	
pJH192	contains the *cro* gene and the intergenic repressor region	This work
	pIV2 D[HindIII-XbaI] W[PCR, 308 bp, HindIII-XbaI], PY54 32,525–32,832	
pJH281	contains 138 bp of the upstream region of the *cI* gene	Figure 6, this work
	pKKluxBamHI D[SmaI-XbaI] W[PCR, 138 bp, SmaI-XbaI], PY54 32,525–32,662	
pJH282	contains 104 bp of the upstream region of the *cI* gene	Figure 6, this work
	pKKluxBamHI D[SmaI-XbaI] W[PCR, 104 bp, SmaI-XbaI], PY54 32,525–32,628	

Table S1. *Cont.*

Plasmids	Description	Reference or Origin
pJH283	contains 80 bp of the upstream region of the *cI* gene<br>pKKluxBamHI D[SmaI-XbaI] W[PCR, 80 bp, SmaI-XbaI], PY54 32,525–32,604	Figure 6, this work
pJH284	contains 59 bp of the upstream region of the *cI* gene<br>pKKluxBamHI D[SmaI-XbaI] W[PCR, 59 bp, SmaI-XbaI], PY54 32,525–32,583	Figure 6, this work
pJH287	contains 64 bp of the upstream region of the *cro* gene including a single nucleotide polymorphism at 1st position of the P1 TATA (-10) box<br>pKKluxBamHI D[SmaI-XbaI] W[PCR, 64 bp, SmaI-XbaI], PY54 32,565–32,628	Figure 6, this work
pJH288	contains 64 bp of the upstream region of the *cro* gene including a single nucleotide polymorphism at 3rd position of the P1 TATA (-10) box<br>pKKluxBamHI D[SmaI-XbaI] W[PCR, 45 bp, SmaI-XbaI], PY54 32,584–32,628	Figure 6, this wor
pJH289	contains 36 bp of the upstream region of the *cro* gene<br>pKKluxBamHI D[SmaI-XbaI] W[PCR, 36 bp, SmaI-XbaI], PY54 32,593–32,628	Figure 6, this work
pJH290	contains 21 bp of the upstream region of the *cro* gene<br>pKKluxBamHI D[SmaI-XbaI] W[PCR, 21 bp, SmaI-XbaI], PY54 32,608–32,628	Figure 6, this work
pJH291	pKKluxBamHI D[SmaI-XbaI] W[PCR, 60 bp, SmaI-XbaI], PY54 32,525–32,584	Figure 6, this work
pJH292	pKKluxBamHI D[SmaI-XbaI] W[PCR, 75 bp, SmaI-XbaI], PY54 32,525–32,599	Figure 6, this work
pJH293	pKKluxBamHI D[SmaI-XbaI] W[SmaI-XbaI, PY54 32,524–32,778]	Figure 7, this work
pJH294	pKKluxBamHI D[SmaI-XbaI] W[SmaI-XbaI, PY54 32,524–32,803]	Figure 7, this work
pJH295	pKKluxBamHI D[SmaI-XbaI] W[SmaI-XbaI, PY54 32,524–32,828]	Figure 7, this work
pJH296	pKKluxBamHI D[SmaI-XbaI] W[SmaI-XbaI, PY54 32,524–32,831]	Figure 7, this work
pJH297	contains an OR3 mutation<br>pKKluxBamHI D[SmaI-XbaI] W[SmaI-XbaI, PY54 32,524–32,831]	Figure 7, this work

Table S2. Purification of the PY54 Cro repressor.

Fraction	Purification Step	Protein [mg] [a]	Recovery [%]	Purity [%]
I	Crude extract [b]	229.2	100	9
II	Heparin-Sepharose™	55.5	24.2	55
III	DEAE-Sephacel™	50.5	22.0	73
IV	CM-Sepharose™ fast flow	34.6	15.1	94

[a] From laser densitometrical measurement on gels; [b] From 29.55 g of cell paste.

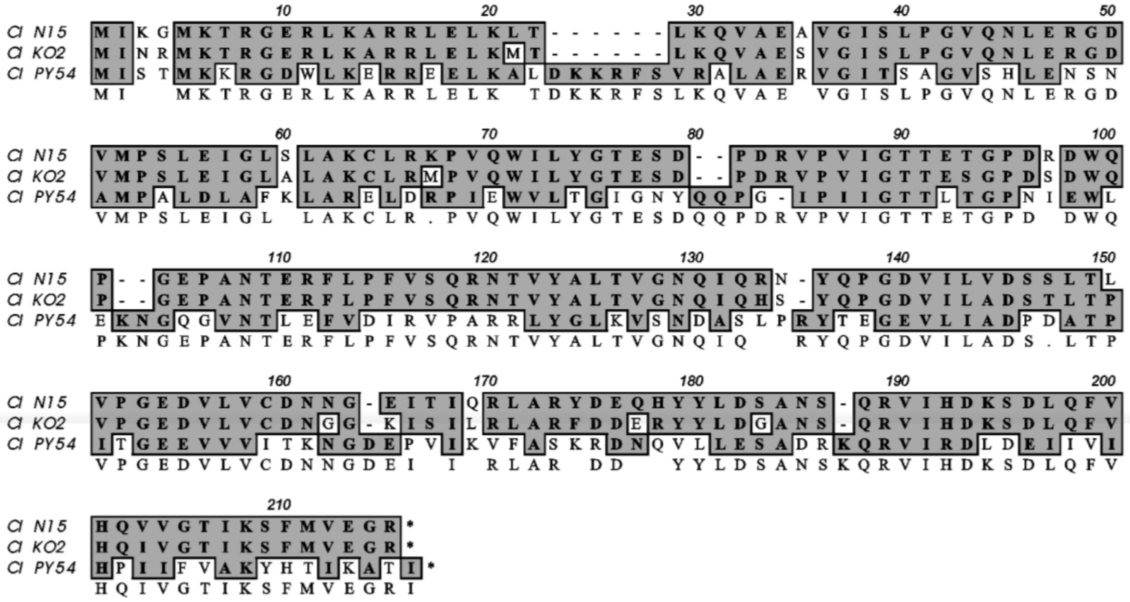

Figure S1. Sequence analysis of PY54 CI. Alignment of the amino acid sequences of the CI/CB prophage repressors of PY54, ϕKO2 and N15. Similar and identical residues at corresponding positions are shown in light grey and dark grey, respectively.

References

1. Sasaki, I.; Bertani, G. Growth abnormalities in Hfr derivatives of *Escherichia coli* strain C. *J. Gen. Microbiol.* **1965**, *40*, 365–376.
2. Hertwig, S.; Klein, I.; Schmidt, V.; Beck, S.; Hammerl, J.A.; Appel, B. Sequence analysis of the genome of the temperate *Yersinia enterocolitica* phage PY54. *J. Mol. Biol.* **2003**, *331*, 605–622.
3. Hertwig, S.; Klein, I.; Appel, B. Properties of the temperate *Yersinia enterocolitica* bacteriophage PY54. *Adv. Exp. Med. Biol.* **2003**, *529*, 241–243.
4. Hertwig, S.; Klein, I.; Lurz, R.; Lanka, E.; Appel, B. PY54, a linear plasmid prophage of *Yersinia enterocolitica* with covalently closed ends. *Mol. Microbiol.* **2003**, *48*, 989–1003.
5. Casjens, S.R.; Gilcrease, E.B.; Huang, W.M.; Bunny, K.L.; Pedulla, M.L.; Ford, M.E.; Houtz, J.M., Hatfull, G.F. Hendrix, R.W. The pKO2 linear plasmid prophage of *Klebsiella oxytoca*. *J. Bacteriol.* **2004**, *186*, 1818–1832.
6. Ravin, N.V. N15: the linear phage-plasmid. *Plasmid* **2011**, *65*, 102–109.

7. Ravin, V., Ravin, N., Casjens, S., Ford, M.E., Hatfull, G.F., Hendrix, R.W. Genomic sequence and analysis of the atypical temperate bacteriophage N15. *J. Mol. Biol.* **2000**, *299*, 53–73.
8. Rybchin, V.N.; Svarchevsky, A.N. The plasmid prophage N15: A linear DNA with covalently closed ends. *Mol. Microbiol.* **1999**, *33*, 895–903.
9. Hammerl, J.A.; Klein, I.; Appel, B.; Hertwig, S. Interplay between the temperate phages PY54 and N15, linear plasmid prophages with covalently closed ends. *J. Bacteriol.* **2007**, *189*, 8366–8370.
10. Covarrubias, L.; Bolivar, F. Construction and characterization of new cloning vehicles. VI. Plasmid pBR329, a new derivative of pBR328 lacking the 482-base- pair inverted duplication. *Gene* **1982**, *17*, 79–89.
11. Balzer, D.; Ziegelin, G.; Pansegrau, W.; Kruft, V.; Lanka, E. KorB protein of promiscuous plasmid RP4 recognizes inverted sequence repetitions in regions essential for conjugative plasmid transfer. *Nucl. Acids Res.* **1992**, *20*, 1851–1858.
12. Strauch, E.; Voigt, I.; Broll, H. Appel, B. Use of a plasmid of a *Yersinia enterocolitica* biogroup 1A strain for the construction of cloning vectors. *J. Biotechnol.* **2000**, *79*, 63–72.
13. Jacob, D.; Lewin, A.; Meister, B.; Appel, B. Plant-specific promoter sequences carry elements that are recognised by the eubacterial transcription machinery. *Transgenic Res.* **2002**, *11*, 291–303.
